# 基于骨髓样本的多发性骨髓瘤微小残留病检测中国专家共识（2024年版）

**DOI:** 10.3760/cma.j.cn121090-20240430-00167

**Published:** 2024-06

**Authors:** 中华医学会血液学分会实验诊断学组、浆细胞疾病学组 中国医师协会多发性骨髓瘤专业委员会

**Keywords:** 多发性骨髓瘤, 微小残留病, 二代流式细胞术, 二代测序, Multiple myeloma, Minimal residual disease, Next-generation flow cytometry, Next-generation sequencing

## Abstract

多发性骨髓瘤（MM）是血液系统第二大常见恶性肿瘤，标准的整体治疗模式及以新药为主的方案极大地改善了MM患者的生存，但微小残留病（MRD）导致大多数患者复发。因此，需要在传统血清学疗效评估的基础上联合MRD检测，以更精准地评估患者的疾病状态。目前，二代流式细胞术（NGF）和二代测序（NGS）是基于骨髓样本检测MRD的主流技术。为使MRD检测标准化和规范化，专家组讨论制订了在MM患者中应用NGF和NGS技术进行骨髓MRD检测的中国专家共识。

## Abstract (en)

Multiple myeloma（MM）is the second most common hematologic malignant tumor. Standard holistic treatment model and new drug-based regimens have greatly improved the survival of patients with MM; however, minimal residual disease（MRD）causes relapse in most patients. Therefore, combining MRD testing on the basis of traditional serological efficacy evaluation is necessary to achieve a more accurate assessment of the patient's disease status. At present, next-generation flow cytometry（NGF）and next-generation sequencing（NGS）are the mainstream technologies for detecting MRD based on bone marrow samples. To standardize and normalize MRD detection, the expert group discussed and formulated Chinese expert consensus on the application of NGF and NGS technology for bone marrow MRD detection in patients with MM.

多发性骨髓瘤（Multiple myeloma，MM）属于浆细胞肿瘤，是血液系统第二大常见恶性肿瘤，约占血液系统肿瘤的10％[1]。2019年中国骨髓瘤年龄标化发病率为0.93/10万，年龄标化死亡率为0.67/10万[2]。标准的整体治疗模式促进了MM患者的规范治疗。蛋白酶体抑制剂、免疫调节剂、达雷妥尤单抗和自体造血干细胞移植的一线使用使多数MM患者获得完全缓解（CR）[3]–[7]。然而即使获得CR，大部分MM患者最终仍会死于疾病复发，提示患者体内存在常规血清学方法无法检测的残留病灶，导致疾病复发。因此需要在常规疗效评估的基础上进行更敏感的微小残留病（Minimal residual disease，MRD）监测，对患者进行治疗反应评价和预后评估。连续MRD监测可以评估不同治疗阶段的疗效[8]。2016年国际骨髓瘤工作组将MRD疗效标准纳入MM诊治指南中[9]，2017年NCCN指南[10]和中国多发性骨髓瘤诊治指南[11]已经将MRD阴性和持续MRD阴性作为疗效判断标准和MM治疗的最好疗效。临床所需的MRD检测技术应能够广泛开展，且具备高度敏感性、高度适用性、价格适宜、检测周期短、使用的样本容量具有可行性等特点。

目前国内各临床中心对于MRD的检测和分析还缺乏统一规范，导致对MM患者MRD检测结果的临床解读和预后评估标准差异极大。为使国内各中心对MRD的检测和分析逐步实现标准化和规范化，尽量减少在结果解读和预后评估标准中的差异，专家组经过讨论形成了基于骨髓样本的MM MRD检测中国专家共识。其中涉及MRD的检测技术主要有二代流式细胞术（Next-generation flow cytometry，NGF）和二代测序（Next-generation sequencing，NGS）[12]–[13]。

一、NGF检测MRD

1. 流式细胞术检测MRD的现状：多参数流式细胞术（Multiparameter flow cytometry，MFC）作为现在使用最广泛的MRD检测手段，具有很多优势：①以异常表型为基础区分正常和异常浆细胞，几乎适用于所有MM患者，不需要初诊结果；②实现了在一个样本中多维分离各种细胞群，对任何异常细胞群进行检测和跟踪；③检测时间短，费用适宜，灵敏度高。

近年来，随着临床治疗的发展及预后判断的需求，NGF被开发运用于检测MRD[14]。NGF技术目前指八色组合以上，同时检测敏感度达到10^−5^及以上的MFC。与传统MFC相比，NGF的灵敏度可达10^−5^～10^−6^，同时具有更高的特异性。但目前国内不同检测中心在抗体组合、荧光搭配、样本处理、圈门策略、分析逻辑、质控管理等诸多技术环节存在差异，导致对于浆细胞肿瘤MRD的检测和分析不规范，对MRD检测结果的临床解读和预后评估标准不一致。为使国内各检测中心NGF逐步实现规范化和标准化，特制定以下标准。

2. NGF检测MRD的抗体选择及常用抗体组合推荐：

（1）检测MRD的抗体选择：CD38和CD138通常用来界定浆细胞群体。所有浆细胞均高表达CD38，但除浆细胞外，CD38在正常B祖细胞、造血祖细胞和成熟单核细胞高表达，在成熟的粒细胞、活化T细胞也有低密度表达，因此仅用CD38设门会造成误差，敏感性强，特异性差，有效率低，不建议单独使用。CD138常表达于浆细胞和一些肿瘤细胞，单独设门圈定浆细胞适用于CD138表达阳性的患者，特异性强，但敏感性低。采用CD38/CD138/CD45设门，联合CD19和CD56可以区分大多数正常和异常浆细胞，浆细胞肿瘤的“骨架”抗体应包含CD38/CD138/CD19/CD56/CD45。正常浆细胞群体CD38（bri）CD138（bri）且非限制性表达胞质免疫球蛋白kappa（Cytosolic immunoglobulin Kappa, cyκ）或lambda（cyλ）轻链（如图1灰色细胞群）。大部分正常浆细胞为CD19（+）CD56（−）CD45（+）CD20（−）CD27（+）CD28（−）CD81（+）CD117（−），符合典型的浆细胞免疫表型特征；小部分正常浆细胞（低于总浆细胞群体的30％）可以为CD19（−）或CD56（+）或CD45（−/low）或CD20（+）或CD27（low），这些非典型的免疫表型一般不会同时出现，最重要的是cyκ/cyλ为非限制性表达。异常浆细胞的免疫表型可以表现为CD38、CD19、CD45、CD27、CD81、CD20、CD28、CD56、CD117的异常表达，且限制性表达轻链cyκ或cyλ（如图1紫色细胞群）。胞质免疫球蛋白轻链的限制性表达是确定浆细胞克隆性最直接的证据，在使用抗体较少时，建议将这两种抗体也纳入骨架抗体中。异常浆细胞还可以表达CD13、CD33等交叉抗原，可视仪器配置情况酌情选用。值得注意的是，部分MM患者治疗后可能出现CD138/CD38的下调甚至转为阴性，初步设门圈定浆细胞群体时要注意结合其他标志。

**图1:**
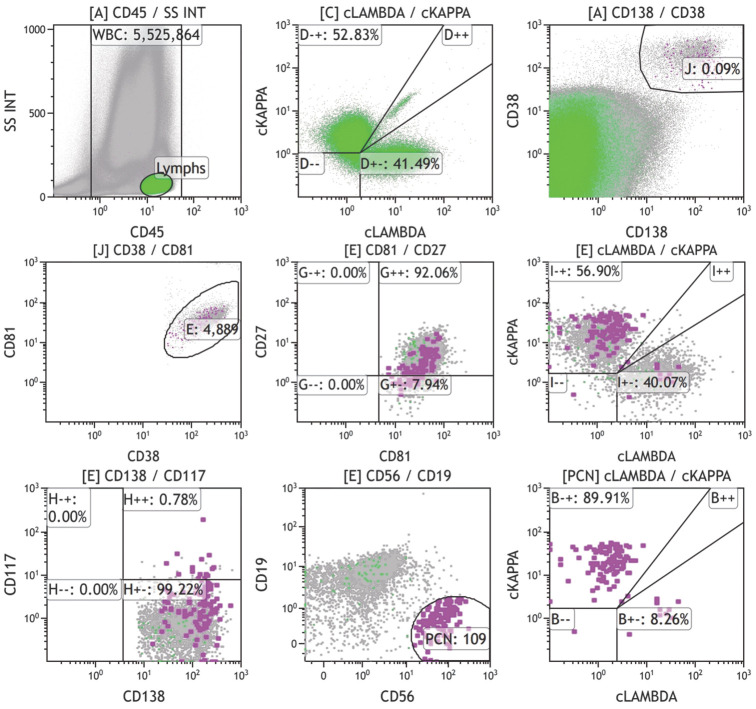
多发性骨髓瘤微小残留病（MRD）报告示例 **注** 分析553万个白细胞，见109个单克隆浆细胞（CD38^++^CD138^++^CD19^−^CD56^+^CD117^−^CD81^+^CD27^±^cKappa^+^cLambda^−^CD45^±^），MRD＝2.0×10^−5^（国际骨髓瘤工作组MRD阳性标准>1.0×10^−5^）；另见8.7×10^−4^正常多克隆浆细胞（CD38^++^CD138^++^CD19^+^CD56^−^CD117^−^CD81^+^CD27^+^cKappa^+^cLambda^+^CD45^+^）（最低定量限＝50/5 525 864＝9.0×10^−6^，最低检出限＝20/5 525 864＝3.6×10^−6^）

（2）浆细胞肿瘤MRD抗体组合推荐：基于目前国内外的报道及国内各大实验室的经验，对检测浆细胞肿瘤的MRD抗体组合进行推荐[15]–[16]（见表1）。其他经过验证的等效组合也可使用。配色方案可根据实验室的具体情况自行设计。

**表1:** 浆细胞肿瘤的微小残留病检测骨架抗体及备选抗体组合推荐表

方法	抗体组合
两管8色	管1：cyκ、cyλ、CD19、CD27、CD138、CD45、CD56、CD38； 管2：CD19、CD27、CD138、CD45、CD56、CD38、CD117、CD81
单管10色	cyκ、cyλ、CD117、CD19、CD138、CD45、CD81、CD38、CD27、CD56

3. 样本前处理及荧光染色、数据获取：样本制备及染色：取3～5 ml骨髓［（5～20）×10^6^个细胞］，加入合适比例的氯化铵溶血剂（具体参照说明书要求）混匀，室温孵育15 min，800×*g*离心10 min弃上清，再加入1×PBS，540×*g*离心5 min洗涤弃上清（重复2次）。洗涤后的细胞悬液（50～100 µl）加入细胞膜标记抗体（如：CD19-ECD/CD117-PC5.5/CD56-PC7/CD138-APC/CD81-APC-A700/CD38-APC-A750/CD27-PB/CD45-KO），混匀后室温避光孵育30 min。加入1×PBS，540×*g*离心5 min洗涤弃上清，振荡混匀。先加入固定剂（具体参照说明书要求），摇匀，室温避光孵育30 min，加入1×PBS，540×*g*离心5 min洗涤弃上清；振荡混匀，加入破膜剂，轻轻摇匀，加入抗体Kappa-FITC/Lambda-PE，混匀，室温避光孵育30 min；加入1×PBS，540×*g*离心5 min洗涤弃上清，加入500 µl PBS重悬细胞，上机检测。每管最少获取>200万个细胞。

4. 流式细胞术MRD数据分析与报告：①初步设门：利用CD45/Time散点图选取液流稳定的区间；利用FSC-Height/FSC-Area和SSC-Height/SSC-Area（或FS-INT/FS-PEAK和SS-INT/SS-PEAK）散点图去除黏连体；利用FSC-Area/SSC-Area散点图去除碎片；先利用特定系列广泛表达的抗原CD45/SSC-Area散点图圈定该系列所有细胞区域，用CD38/CD138散点图圈定所有浆细胞群体。②进一步分析MRD：用CD38/CD138散点图圈定浆细胞群体，根据CD38、CD19、CD45、CD27、CD81、CD20、CD28、CD56、CD117的表达水平圈定异常浆细胞，分析轻链cyκ和cyλ的表达，计算出单克隆浆细胞的数量（图1紫色细胞群）。③计算MRD水平：计算MRD定量结果的方法为异常单克隆浆细胞占所有白细胞的百分比。当异常浆细胞不表达CD45时，白细胞数量应包含CD45阳性白细胞和阴性区的异常浆细胞。④报告内容：MRD结果报告为“阳性”或“阴性”。MRD如果为阳性，应描述报告所见异常细胞数量及异常细胞占白细胞比例，并描述异常细胞的免疫表型。报告获取的白细胞总数，同时报告方法学最低检出限（Limit of detection，LOD）和（或）最低定量限（Lower limit of quantification，LLOQ），国际上的研究认为20个细胞是LOD比较保守的值。如果预期敏感性需要达到1×10^−5^水平，至少要获取200万个细胞；而LLOQ是指可重复定量检出MRD阳性细胞群体的最小细胞数，推荐可定量肿瘤细胞群体的最小细胞数为50个，如果预期敏感性要达到1×10^−5^水平，至少要获取500万个细胞。报告中应出具一系列散点图，显示异常（亮色突出）和正常细胞群体的抗原表达及散射光信号。

靶向CD38的单克隆抗体如达雷妥尤单抗和艾沙妥昔单抗（Isatuximab）已广泛用于MM治疗，但此类单抗药物会干扰流式细胞术对浆细胞CD38的识别，导致假阴性结果。因此，建议留意应用此类药物患者CD138^+^CD38^−^细胞群或使用多表位CD38Me、CD38纳米单抗或使用可识别内质网膜上CLIMP-63蛋白的VS38c等代替常规CD38来识别浆细胞。

二、NGS技术的MRD检测

1. NGS技术检测MRD：NGS技术检测MRD主要指通过设计多种引物PCR扩增结合深度测序（LymphoSIGHT平台[17]或经等效性验证的技术方法[18]），检测患者全骨髓液中肿瘤性浆细胞的免疫球蛋白基因片段（IGH-VDJH、IGH-DJH或IGK）重排克隆[19]。患者缓解期MRD检测必须有基线样本克隆信息，基线样本的目的是确定肿瘤性浆细胞的克隆性重排序列，即鉴定显著性克隆，以用于后续MRD检测。因此，理想的基线样本应来自初诊未治疗或复发未治疗患者[20]；在上述样本无法获取的情况下，也可使用来自肿瘤负荷比较高的某一治疗时间点的储存样本定义基线状态。推荐基线样本克隆鉴定和缓解样本MRD检测在同一家实验室，或通过室间比对的同等实验室进行[18]。

2. 实验检测流程：

①样本采集：推荐采集新鲜骨髓，保存在EDTA或枸橼酸钠抗凝的采血管，禁用肝素抗凝。如需外送，采血管4 °C冷藏运送不超过3 d，也可使用冻存骨髓、细胞或DNA。对于缓解患者，骨髓采集量以3～5 ml为宜，若白细胞计数偏低，应适当调整采集量，使有核细胞总数达到2×10^6^个以上。②核酸制备：提取满足实验要求、质量合格的DNA样本，根据不同MRD检测灵敏度加入合适的DNA起始样本量。③文库制备和上机：以IonTorrent测序平台为例，文库制备和上机检测基本流程见图2。④靶标基因选择：选择特异性好的克隆作为MRD随访标志物，部分重排类型在人群中出现概率较高，会导致假阳性结果，需慎重选择。⑤灵敏度严谨：灵敏度由样本投入量和测序数据量共同决定，必须达到实验室宣称的灵敏度才有意义，否则可能是假阴性结果[21]。国际骨髓瘤工作组（IMWG）和中国多发性骨髓瘤诊治指南共同推荐MRD检测灵敏度需达到10^−5^及以上[9]。

**图2:**
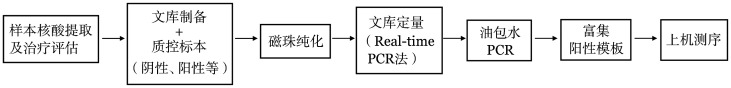
二代测序检测微小残留病实验基本流程

3. 生物信息学分析流程：

（1）下机后的测序数据需要进行质量评估，包括测序数据质量、测序覆盖度、阴性和阳性标本检测结果等评价参数。

（2）需设定明确的测序数据过滤标准，对过滤后的合格序列使用NCBI、IMGT等数据库进行克隆序列比对[22]，也可使用商品化试剂配套的一站式分析流程软件[23]。

（3）基线样本中肿瘤细胞克隆性重排的鉴定标准为：①测序结果中至少2条完全相同的序列定义为克隆性重排序列。②应用目前已有的克隆性重排判断金标准，如Sanger测序法、毛细管电泳（CE）方法等。③根据各实验方法建立的判定规则来定义[24]。④缓解期样本MRD阳性的定义标准为：缓解期检测到的克隆序列与基线克隆序列一致，则为检测到肿瘤细胞MRD；新出现的显著性克隆建议具体分为2种情况：原有克隆的演化克隆如V区替换、重排后突变等[25]；原有非显著性克隆（基线时存在但未达显著性克隆判定标准），对于上述克隆建议复查随访进一步确认其临床病理意义。

4. 报告单内容：

（1）报告本次检测的DNA上样总量。

（2）选取的目标基因。

（3）克隆性重排的类型和比例。

（4）MRD结果阳性或阴性。如报告结果为MRD阴性，建议使用术语uMRD（未检测到MRD）；如报告结果为MRD阳性，建议提供可检测MRD的定量结果[26]。目前MRD定量水平报告的计算方式可以有两种：①以肿瘤细胞克隆性重排序列数占总B细胞重排序列数的比例表示；②以肿瘤细胞克隆细胞数占总有核细胞数的比例表示[25]。

（5）特别强调的是，如果MRD检测结果为阴性，应具体说明该标本此次MRD检测结果在相应灵敏度的置信区间。

（6）如患者有2次及以上MRD数据，可根据MRD水平和检测时间点，绘制相应的检测点曲线，以动态监测MRD水平的变化（如图3）。

**图3:**
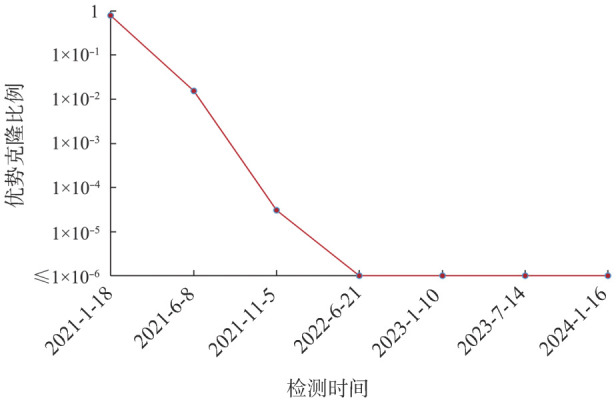
二代测序监测1例多发性骨髓瘤患者微小残留病动态变化

其余NGS检测MRD实验流程、生物信息学分析、质量控制和管理、报告单内容等推荐参考行业要求和规范[9],[21],[27]–[29]。

三、MRD检测的临床意义及方法选择

1. 检测的时间点和频率：MRD检测需要在常规疗效评估基础上进行。一般在常规疗效评估可能达CR时开始进行MRD检测[9]。

在临床研究规定的治疗方案中，根据研究目的，推荐在诱导治疗结束后进行第一次MRD评估；对于接受移植和巩固治疗的患者，推荐在移植后3个月或巩固治疗结束后进行MRD检测，确认移植和巩固治疗疗效；维持治疗阶段，MRD检测未转阴患者每半年1次，转阴的患者可以至少每年检测1次。观察患者的MRD动态变化和持续性直至确认复发或进展。

未接受移植的患者可以在疗效达CR时，或固定疗程结束后行MRD检测，MRD检测未转阴患者每半年1次，转阴的患者可以至少每年检测1次，直至确认复发或进展[30]。

2. MRD检测的阈值：最新的IMWG指南建议基于骨髓样本的检测最小灵敏度要达到10^−5^或更高。各中心确定有临床意义的阈值必须验证与临床预后具有很好的相关性。

3. 方法的比较：NGF重要优点是高适用性（接近100％）、检测时间短（2 h）、可以普遍开展。其敏感性较前明显提高，达到10^−5^～10^−6^，同时可以区分正常浆细胞与异常浆细胞。流式细胞术MRD的检测不依赖患者初诊资料。但由于没有评估肿瘤特异性抗原，容易在检测中遗漏异常表型。流式细胞术要求标本新鲜，要求临床及时送检。

NGS也具有普遍适用性（>90％），灵敏度达10^−5^～10^−6^。国内目前有商业化的配套试剂和一站式的生物信息学分析流程，易于实现技术标准化，可以获取治疗过程中的克隆演化信息且存储的标本也可以使用[14]。缺点是需要基线样本鉴定显著性克隆，详见表2。

**表2:** 二代流式细胞术（NGF）与二代测序（NGS）特征比较

特征	NGF	NGS
适用性	接近100%	>90%
检测时间	短，2 h	较长，5～7 d
标本要求	高，必须新鲜细胞	低，新鲜、冻存均可
灵敏度	10^−5^～10^−6^	10^−5^～10^−6^
标准化	不易	较容易

4. MRD的临床意义：目前国内外已有大量文献报道MRD与MM患者预后的关系。大宗临床研究数据（表3）显示，无论是移植还是非移植患者，无论是否具有遗传学高危因素，获得MRD阴性、尤其是持续MRD阴性患者的预后明显优于MRD阳性患者[31]–[32]，因此要注意在不同时间点连续进行MRD监测，并结合PET-CT等影像学检查。目前为止，已经有一些临床研究根据MRD水平进行治疗决策，已经有个别临床研究进行数据披露。但是临床治疗中基于MRD阳性而不是阴性做出决策可能更加安全。若将MRD的监测作为临床制定治疗策略的标准，还需在临床研究中证实（表4）。

**表3:** 部分进行二代微小残留病（MRD）监测的多发性骨髓瘤临床研究数据

研究	方案	中位无进展生存（PFS）	中位总生存	MRD检测方法（灵敏度）	MRD阴性率
适合移植					
IFM2009（NCT01191060）	HDT对RVD	HDT：50个月， RVD：36个月	HDT：NR， RVD：NR	NGS（10^−6^）	HDT：30%， RVD：20%
CASSIOPEIA（NCT02541383） （第1部分）	D-VTd对VTd	D-VTd：18个月PFS 92.7%， VTd：18个月PFS 84.6%	D-VTd：NR， VTd：NR	NGF（10^−5^）	D-VTd：64%， VTd：44%
CASSIOPEIA（NCT02541383） （第2部分）	D维持治疗对观察	D维持治疗：NR， 观察：46.7个月	D维持：NR， 观察：NR	NGF（10^−5^）	D维持：58.6%， 观察：47.1%
不适合移植					
ALCYONE（NCT02195479）	D-VMP对VMP	D-VMP：36.4个月， VMP：19.3个月	D-VMP：NR， VMP：NR	NGS（10^−5^）	D-VMP：22.3%，VMP：6.2%
CLARION（NCT01818752）	KMP对VMP	KMP：22.3个月， VMP：22.1个月	KMP：NR， VMP：NR	NGF（10^−6^）	KMP：15.7%，VMP：15.5%
MAIA（NCT02252172）	DRd对Rd	DRd：61.9个月， Rd：34.4个月	DRd：NR， Rd：65.5个月	NGS（10^−5^）	DRd：32.1%， Rd：11.1%
复发难治					
POLLUX（NCT02076009）	DRd对Rd	DRd：69.3个月， Rd：23.1个月	DRd：67.6个月， Rd：51.8个月	NGS（10^−5^）	DRd：33.2%， Rd：6.7%
CASTOR（NCT02136134）	DVd对Vd	DVd：25.4个月， Vd：9.7个月	DVd：49.6个月， Vd：38.5个月	NGS（10^−5^）	DVd：15.1%， Vd：1.6%

**注** HDT：大剂量美法仑和自体造血干细胞移植；RVD：来那度胺+硼替佐米+地塞米松；D-VTd：达雷妥尤单抗+硼替佐米+沙利度胺+地塞米松；D：达雷妥尤单抗；D-VMP：达雷妥尤单抗+硼替佐米+美法仑+泼尼松；KMP：卡非佐米+美法仑+泼尼松；DRd：达雷妥尤单抗+来那度胺+地塞米松；DVd：达雷妥尤单抗+硼替佐米+地塞米松；NR：未达到；NGS：二代测序；NGF：二代流式细胞术

**表4:** 部分以微小残留病（MRD）指导多发性骨髓瘤治疗策略的临床研究

国家临床试验（NCT）识别号	研究名称	国家	技术
NCT02406144	新诊断症状性多发性骨髓瘤患者自体造血干细胞移植后使用来那度胺和地塞米松维持治疗与来那度胺、地塞米松和伊沙佐米的维持治疗——以MRD状态为指导的维持时间（GEM2014MAIN）	西班牙	NGF
RADAR	根据疗效指导的风险分层治疗比较新诊断的适合造血干细胞移植的多发性骨髓瘤患者的治疗升级和降级策略	英国	N/A
NCT03490344	诱导治疗后有/无大剂量化疗/自体造血干细胞支持的MRD阳性骨髓瘤患者短期使用达雷妥尤单抗	美国	FC
NCT03224507	为达多发性骨髓瘤深度缓解的基于单克隆抗体的序贯疗法（MASTER）	美国	NGS
NCT03742297	硼替佐米-美法仑和泼尼松（VMP）诱导治疗，随后来那度胺和地塞米松（Rd）维持治疗对比卡非佐米、来那度胺和地塞米松（KRd）±达雷妥尤单抗（18个周期）随后来那度胺和达雷妥尤单抗巩固和维持治疗：一项针对年龄65岁至80岁之间的新诊断多发性骨髓瘤老年患者的3期、多中心、随机研究	西班牙	NGF
NCT03697655	达雷妥尤单抗抢先治疗MRD复发或生化复发的多发性骨髓瘤（PREDATOR）	波兰	N/A
NCT03710603	达雷妥尤单抗、硼替佐米、来那度胺和地塞米松（D-VRd）与硼替佐米、来那度胺和地塞米松（VRd）治疗既往未经治疗且符合高剂量治疗条件的多发性骨髓瘤患者的3期临床研究（PERSEUS）	欧洲骨髓瘤网络	N/A
NCT03992170	达雷妥尤单抗治疗疗效达VGPR以上且二代流式细胞术MRD阳性的多发性骨髓瘤患者的初步研究（DART4MM）	意大利	FC
NCT02969837	埃罗妥珠单抗、卡非佐米、来那度胺和低剂量地塞米松（E-KRd）初始治疗新诊断的需要全身化疗的多发性骨髓瘤的开放标签、单臂、2期研究	美国	NGS和MFC
NCT04071457	多发性骨髓瘤患者自体造血干细胞移植后应用达雷妥尤单抗/重组人透明质酸酶（rHuPH20）（NSC-810307）+来那度胺或来那度胺维持治疗并基于MRD指导治疗持续时间的3期研究	美国	NGS
NCT04096066	比较卡非佐米-来那度胺-地塞米松（KRd）与来那度胺-地塞米松（Rd）治疗不适合自体造血干细胞移植的新诊断骨髓瘤患者的随机3期研究	意大利	N/A
NCT03376477	来那度胺联合同种异体骨髓瘤粒细胞巨噬细胞刺激因子（GM-CSF）疫苗治疗完全或接近完全缓解的多发性骨髓瘤患者的随机、双盲、安慰剂对照的2期研究	美国	NGS
NCT04108624	MRD检测的多模式方法指导多发性骨髓瘤移植后维持治疗（MRD2STOP）	美国	NGS
NCT04221178	针对持续MRD阴性缓解的多发性骨髓瘤患者停止维持治疗的单臂前瞻性研究	美国	NGF
NCT04140162	在多发性骨髓瘤患者中根据MRD驱动不同基于达雷妥尤单抗的前线治疗的2期研究	美国	N/A

**注** NGF：二代流式细胞术；N/A：不详；FC：流式细胞术；NGS：二代测序；MFC：多参数流式细胞术；VGPR：非常好的部分缓解
